# Influence of Posture, Spinal Level, Gender and Muscle Activation on Biomechanical Properties of Lumbar Erector Spinae in Healthy Young Adults

**DOI:** 10.3390/medicina62010159

**Published:** 2026-01-13

**Authors:** Yueh-Ling Hsieh, Heng-Yi Lin, Andy Chien

**Affiliations:** 1 Department of Physical Therapy, China Medical University, Taichung 40402, Taiwan; sherrie@mail.cmu.edu.tw (Y.-L.H.); u114030017@cmu.edu.tw (H.-Y.L.); 2 Department of Biomedical Engineering, College of Medicine and College of Engineering, National Taiwan University, Taipei 10617, Taiwan

**Keywords:** lumbar erector spinae, muscle biomechanical properties, stiffness, tone, damping, posture, gender, spinal level, paraspinal muscle assessment

## Abstract

*Background and Objectives*: This study set out to better understand how posture, spinal level, gender and muscle activation influence the biomechanical properties of the lumbar erector spinae (LES) in healthy young adults. We aimed to measure how these factors influence LES tone, stiffness, and damping using a myotonometry device. *Materials and Methods*: Thirty healthy young adults (14 males, 16 females; aged 20–25 years) were evaluated at bilateral L3–L5 levels in prone, unsupported sitting, and standing positions, both under relaxed conditions and during submaximal isometric lumbar extension. The myotonometer measured LES tone (Hz), stiffness (N/m), and damping (logarithmic decrement). For each outcome, a mixed-model repeated-measures ANOVA was conducted with Gender as a between-subject factor and Posture, Level, and Action (relaxed vs. contracted) as within-subject factors (Bonferroni-adjusted α = 0.0167). *Results*: Posture produced the most significant and consistent effects on all properties—stiffness, tone, and damping (*p* < 0.0167)—with sitting and standing generally increasing stiffness and tone compared to prone, and sitting showing the highest values. Gender significantly impacted stiffness and tone (*p* < 0.0167), with males showing higher values. Spinal level also significantly influenced damping, stiffness, and tone (all *p* < 0.0167), with differences more apparent in females. Significant interactions included the influence of Posture × Gender on tone and damping (*p* < 0.0167), and of Posture × Action on stiffness and tone (*p* < 0.0167), alongside a strong three-way interaction for Level × Action × Posture across all outcomes, suggesting posture-related responses depend on activation state and spinal level. *Conclusions*: LES biomechanical properties are strongly affected by posture and further modulated by muscle activation, gender, and spinal level. These results support the creation of posture- and gender-specific reference values and underscore the value of dynamic, posture-specific myotonometer-based assessments for paraspinal muscle evaluation and clinical planning.

## 1. Introduction

The lumbar erector spinae (LES) muscle is critical for maintaining spinal stability, controlling posture, and facilitating trunk movement, making it essential for lumbar motion and postural control [1]. Previous studies have demonstrated that the biomechanical and mechanical properties of lumbar paraspinal muscles vary according to posture, spinal level, and muscle activation demands, reflecting differences in local loading conditions and functional roles across segments [2,3]. However, excessive activation of the LES due to factors such as low back pain can result in reduced lumbar range of motion, delayed movement, reduced movement variability for spinal load distribution, muscle fatigue, and other issues [1,4]. Understanding the intrinsic biomechanical properties of the LES, specifically damping, stiffness, and tone, is therefore crucial for clinical applications in prevention, assessment, and rehabilitation. These properties, which reflect the muscle’s resistance to movement and its resting tension, are highly sensitive indicators of neuromuscular function and underlying pathology, such as chronic low back pain [4,5]. However, accurately measuring these properties is complicated by their known dependency on dynamic physiological conditions (e.g., posture changes, fatigue, or tension levels), with additional variability potentially related to biological sex [6,7,8].

The handheld myotonometer is a widely used, non-invasive, portable device that quantifies the biomechanical and viscoelastic properties of superficial muscles and soft tissues. By delivering a brief, high-precision mechanical impulse to the muscle belly, it induces damped oscillations from which tone, stiffness, and the logarithmic decrement are derived [9]. Robust inter- and intra-rater reliability has been demonstrated across multiple muscle groups, including the paraspinal muscles [10,11]. Such measurement properties have supported the application of myotonometer in athletic, occupational, and physically active populations to evaluate changes in muscle mechanical properties associated with mechanical loading, posture-related demands, ergonomic exposure, physical performance, and recovery interventions [12,13,14,15,16]. Previous findings further indicate that muscle mechanical properties assessed by myotonometry are not static but vary according to age, gender, muscle activation state (relaxed vs. contracted), and spinal level [17,18]. However, most existing studies have examined muscle mechanical properties under limited or singular conditions, without systematically considering the combined influence of posture, spinal level, and muscle activation. Posture is also a key modulator, as it markedly alters the passive and active mechanical loading of the LES, thereby influencing its stiffness and tone [19]. Although resting-state measures are reliable, values obtained solely from relaxed, static postures may not accurately represent the mechanical demands encountered during movement. In contrast, posture-induced changes in muscle mechanical properties may more closely relate to the pain experienced during functional activities [17].

Despite this evidence, comprehensive multifactorial studies that simultaneously analyze the combined and interactive effects of posture, gender, muscle activation state, and spinal level on LES biomechanical properties remain scarce. Therefore, this study aimed to characterize how these key factors collectively influence the intrinsic biomechanical properties (tone, stiffness, and damping) of the LES in healthy young adults, using a myotonometry-based protocol across prone, sitting and standing postures.

## 2. Materials and Methods

### 2.1. Participants

An a priori power analysis (G*Power, version 3.1.9.7; Heinrich Heine University, Düsseldorf, Germany) showed that a sample size of 30 participants achieved approximately 84% statistical power to detect medium-sized effects (Cohen’s f = 0.25, α = 0.05). A total of 30 healthy adult volunteers (14 males, 16 females) aged 20–25 years were randomly recruited from the university student population. Inclusion criteria required participants to no palpable taut bands or tenderness in their lower back muscles, but no history of major low back pain episodes or diagnosed spinal, hip, or pelvic diseases. Exclusion criteria included individuals with symptoms of radiculopathy, trauma-induced lower back pain, diagnosed spinal deformities (e.g., scoliosis or spondylolisthesis), lumbar disc herniation, spinal infections or tumors, rheumatic diseases, a history of spinal surgery, or a treated history of hip or pelvic disorders. Furthermore, individuals with a Body Mass Index (BMI) of 30 kg/m^2^ or higher (as defined by standardized myotonometry guidelines) were also excluded. All participants provided written informed consent prior to their involvement. The study protocol adhered to the ethical principles of the Declaration of Helsinki and was approved by the Institutional Review Board of China Medical University Hospital (No.: CMUH110-REC2-071, 10 May 2021).

### 2.2. Myometric Parameter Measurement

**Figure 1:**
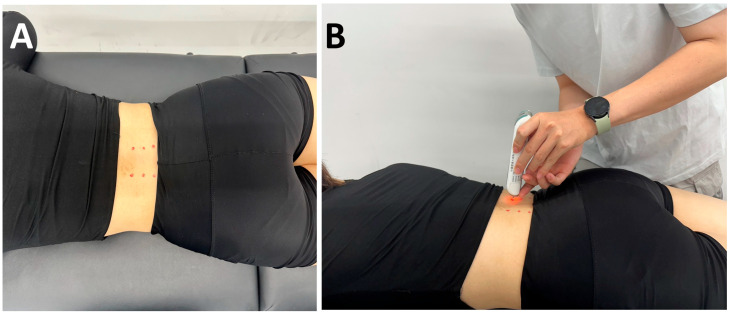
(**A**) Bilateral measurement points were identified and marked 2–3 cm lateral to the L3–L5 spinous processes. (**B**) MyotonPRO assessments were then performed sequentially at six predefined sites spanning the L3–L5 levels.

To ensure consistency and the fixation of measurement points, participants were positioned in a relaxed prone position, and the bilateral LES were palpated and marked at approximately 2–3 cm lateral to the spinous processes of the L3, L4, and L5 vertebrae (Figure 1A). The primary myometric properties assessed were tone, stiffness, and damping in LES. These mechanical properties were assessed bilaterally on the LES of all participants using a small, non-invasive handheld myotonometer (MyotonPRO, Myoton Ltd., Tallinn, Estonia). The probe was positioned perpendicularly and stably on the skin surface over the LES muscle bellies. A constant preload of 0.18 N was first applied to compress the subcutaneous tissue, followed by a 15-ms mechanical impulse of 0.4 N, which elicited a damped natural oscillation within the muscle. The resulting oscillatory response was recorded as an acceleration signal, which was automatically analyzed by the device software to determine muscle tone (frequency, Hz) and dynamic stiffness (N/m). Damping was quantified by the logarithmic decrement of the damped oscillations, reflecting the tissue’s ability to restore its shape after deformation.

Measurements were taken sequentially on the left and right sides of the body, covering the L3, L4, and L5 regions at six predefined points (Figure 1B). Each site was tested using a mode of three mechanical impulses, yielding three independent readings that were averaged for subsequent analysis. Previous studies have demonstrated excellent reliability of the MyotonPRO (ICC > 0.93) across all measurement modes, including both single and averaged triple compressions used in this study [20]. Moreover, the device showed higher test–retest reliability than tensiomyography when assessing the erector spinae at the L3–L4 level in healthy adults [21].

### 2.3. Experimental Procedures

**Figure 2:**
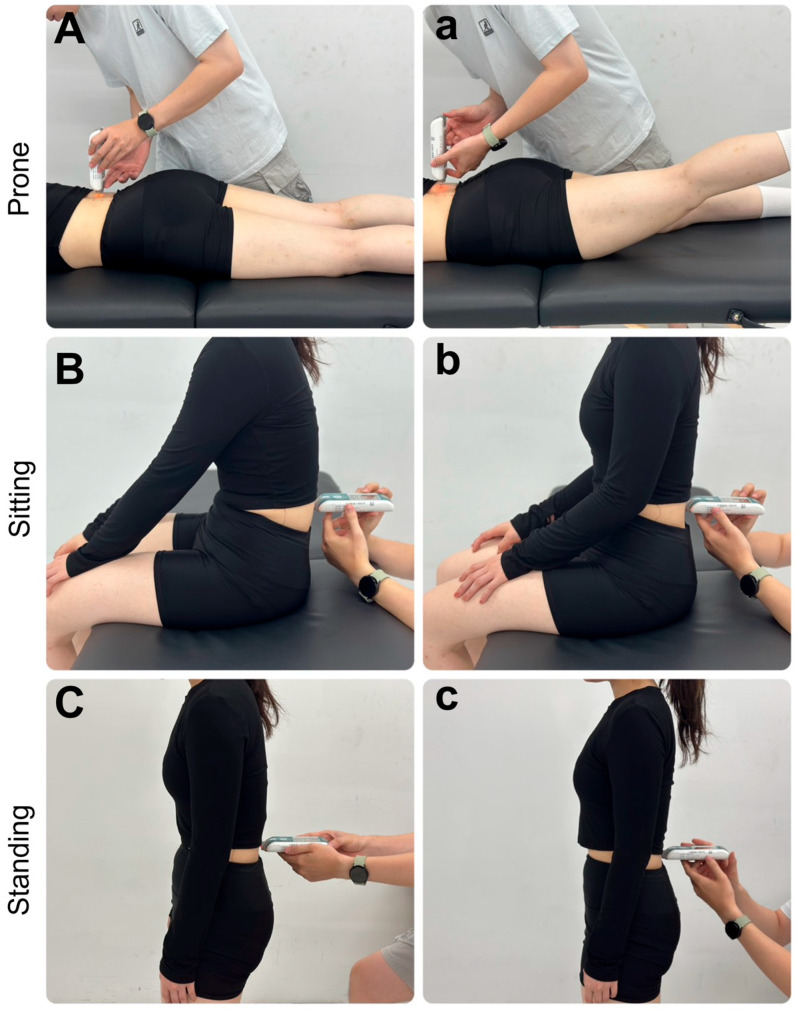
MyotonPRO assessments of the lumbar erector spinae were performed in three postures—prone, unsupported sitting, and standing—under relaxed (**A**–**C**) and contracted (**a**–**c**) conditions at the L3–L5 levels.

This study employed a cross-sectional observational design to investigate the effects of different genders, measurement levels, postures, and muscle actions on the biomechanical properties of the LES. The testing postures were performed in a sequential order: prone, unsupported sitting, and static standing. In each posture, the MyotonPRO device was used to measure the muscle properties of the LES at bilateral sides of L3, L4 and L5 levels during both a relaxed state and a contracted state (Figure 2). During contraction trials, participants were instructed to perform a submaximal isometric lumbar extension corresponding to approximately 30–40% of their perceived maximal effort. To standardize the subjective contraction intensity, this effort was described as a moderate, clearly perceptible contraction that was noticeably greater than postural activation but well below maximal exertion, and could be maintained comfortably without breath holding or discomfort, while avoiding compensatory movements of the pelvis or lower limbs. A brief familiarization period preceded data collection to ensure consistent performance.

### 2.4. Statistical Analysis

The normality of data distribution for each variable was assessed using the Shapiro–Wilk test (*p* > 0.05). Prior to the main statistical analyses, no significant differences were found between values obtained from the left and right sides at each vertebral level (L3, L4, L5) for any parameter (paired *t*-tests, *p* > 0.05). Therefore, to yield a single mean value for each participant, the raw values for tone, stiffness, and damping at each spinal level were averaged across the two sides. This process yielded one mean measurement value for each of the three spinal segments (L3, L4, and L5) per participant. Subsequently, descriptive statistics (mean ± standard deviation, SD) were calculated for all demographic and myometric variables. For each outcome (damping, stiffness, tone), a mixed-model repeated-measures ANOVA was performed with Gender (male vs. female) as the between-subject factor and Posture (prone, sitting, standing), Level (L3, L4, L5), and Action (relaxed vs. contracted) as within-subject factors. Partial eta-squared (η^2^) values were reported to indicate effect sizes. A post hoc power analysis was performed using G*Power software to provide an estimate of statistical power based on the observed effect sizes and final sample size; the estimated power for the primary outcomes exceeded 0.90. The mixed-model repeated-measures ANOVA performed on the three primary outcomes (damping, stiffness, and tone) served as the primary inferential analysis. Accordingly, a Bonferroni correction was applied to adjust the overall significance level from α = 0.05 to α = 0.0167 (0.05/3) for the evaluation of main and interaction effects, thereby controlling the family-wise Type I error rate. Following significant omnibus findings, post hoc analyses were performed to identify specific differences between postures, spinal levels, and muscle contraction states within each gender. Postural comparisons were conducted using one-way ANOVA followed by Dunnett’s post hoc test, with the prone posture serving as the reference condition under the same muscle action state. Differences between muscle relaxation and contraction were examined using paired *t*-tests within the same posture and spinal level. Unless otherwise specified, the nominal significance level for post hoc analyses was set at α = 0.05. All statistical analyses were performed using SPSS software (version 22.0; IBM Corp., Armonk, NY, USA).

## 3. Results

### 3.1. Demographic Characteristics

Thirty healthy adults participated (16 females, 14 males, Table 1). Females averaged 21.06 ± 2.02 years, 51.88 ± 5.03 kg, and 1.59 ± 0.06 m (BMI 20.64 ± 1.73), whereas males averaged 21.21 ± 1.85 years, 66.93 ± 10.03 kg, and 1.74 ± 0.05 m (BMI 22.01 ± 2.47).

### 3.2. Global Effects of Gender, Posture, Level, and Action

Mixed-model repeated-measures ANOVA showed that the biomechanical properties of the LES were predominantly influenced by Posture and Level, followed by Gender and several higher-order interactions (Table 2). In contrast, Action (relaxed vs. contracted) did not demonstrate a significant main effect on any outcome (all *p* > 0.0167).

For damping, Gender did not show a significant effect (F(1, 28) = 0.32, *p* = 0.58, η^2^ = 0.01). Significant main effects were observed for Posture (*p* < 0.0167, η^2^ = 0.28), along with a significant Posture × Gender interaction (*p* < 0.0167, η^2^ = 0.19). A significant main effect was also found for Level (*p* < 0.0167, η^2^ = 0.36), together with a significant Level × Posture interaction (*p* < 0.0167, η^2^ = 0.18). Additionally, significant three-way interactions were detected for Level × Action × Gender (*p* < 0.0167, η^2^ = 0.20) and Level × Action × Posture (*p* < 0.0167, η^2^ = 0.19).

For stiffness, Gender was significant (F(1, 28) = 14.27, *p* < 0.0167, η^2^ = 0.34). Significant main effects were found for Posture (*p* < 0.0167, η^2^ = 0.70), while Posture × Gender was not significant (*p* = 0.41, η^2^ = 0.03). A significant main effect was also found for Level (*p* < 0.0167, η^2^ = 0.43), along with a significant Level × Action × Posture interaction (*p* < 0.0167, η^2^ = 0.24). The main effect of Action did not reach significance (*p* = 0.69, η^2^ = 0.01), but significant interactions were observed for Action × Posture (*p* < 0.0167, η^2^ = 0.59) and Action × Posture × Gender (*p* < 0.0167, η^2^ = 0.23).

For tone, Gender was significant (F(1, 28) = 9.95, *p* < 0.0167, η^2^ = 0.26). Significant main effects were found for Posture (*p* < 0.0167, η^2^ = 0.63), along with a significant Posture × Gender interaction (*p* < 0.0167, η^2^ = 0.18). A significant main effect was also found for Level (*p* < 0.0167, η^2^ = 0.61), together with a significant Level × Action × Posture interaction (*p* < 0.0167, η^2^ = 0.33). The main effect of Action again showed no significance (*p* = 0.66, η^2^ = 0.01), whereas Action × Posture was significant (*p* < 0.0167, η^2^ = 0.53).

### 3.3. Posture Effects Stratified by Gender and Muscle State

**Figure 3:**
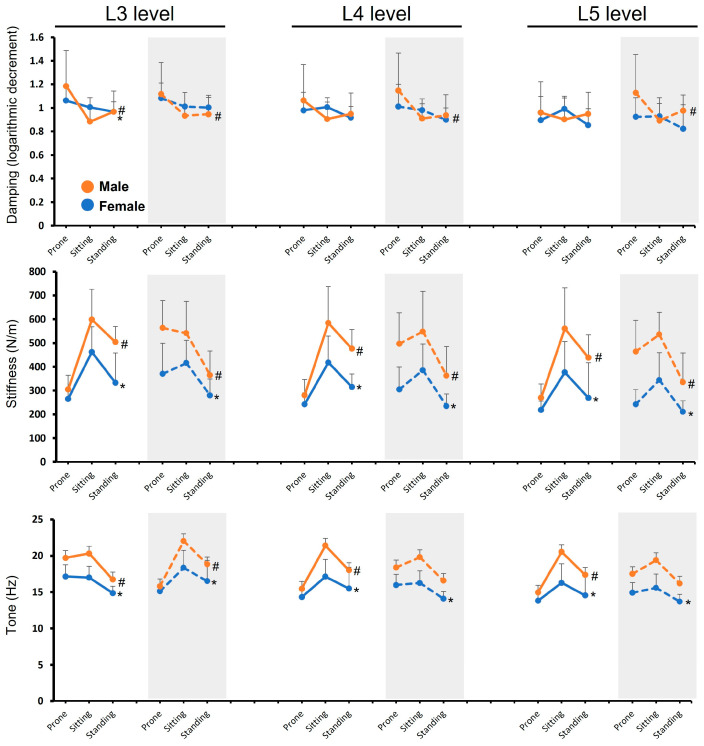
Damping, stiffness, and tone of the lumbar erector spinae at the L3, L4, and L5 levels across three postures (prone, sitting, and standing) in male (orange) and female (blue) participants. White panels indicate measurements obtained under a relaxed muscle condition, whereas grey-shaded panels indicate measurements under a contracted muscle condition. Data are presented as mean ± SD. #, *p* < 0.05, denotes significant differences among postures within the male group; *, *p* < 0.05, denotes significant differences among postures within the female group.

One-way ANOVA was conducted to compare LES biomechanical properties at spinal levels L3–L5 across the three postures (prone, sitting, and standing) separately for relaxation and contraction, and stratified by gender (Table 3, Figure 3). For damping, posture significantly affected female damping during relaxation at L3 (F = 3.49, *p* = 0.039). Post hoc analysis (Dunnett’s test) indicated that standing posture resulted in significantly lower damping at L3 than the prone posture (*p* < 0.05). The posture effect was non-significant for females at L4 (*p* = 0.08) and L5 (*p* = 0.05) during relaxation, and at all levels during contraction (L3: *p* = 0.13, L4: *p* = 0.06, L5: *p* = 0.13). For males, posture significantly influenced damping during relaxation at L3 (F = 7.35, *p* = 0.002), with post hoc testing showing that both sitting and standing postures resulted in significantly lower damping at L3 than prone (*p* < 0.05). The effect of posture was non-significant at L4 (F = 1.98, *p* = 0.152) and L5 (F = 0.29, *p* = 0.752) during relaxation. Posture also significantly affected male damping during contraction across all levels: L3 (F = 4.44, *p* = 0.018), L4 (F = 4.77, *p* = 0.014), and L5 (F = 3.77, *p* = 0.032). In the contracted state, both sitting and standing resulted in significantly lower damping at L3 and L4 compared to prone (*p* < 0.05); however, only the sitting posture resulted in significantly lower damping at L5 compared to prone (*p* < 0.05).

Posture exerted a highly significant influence on stiffness for females across all levels during both relaxation (L3: F = 16.91, *p* < 0.001; L4: F = 9.79, *p* < 0.001; L5: F = 7.74, *p* = 0.001) and contraction (L3: F = 7.58, *p* = 0.001; L4: F = 11.58, *p* < 0.001; L5: F = 12.37, *p* < 0.001). Post hoc analysis showed that sitting consistently resulted in significantly higher stiffness at L3, L4, and L5 than prone during relaxation (*p* < 0.05). During contraction, sitting maintained significantly higher stiffness at L4 and L5 than prone (*p* < 0.05), whereas standing at L3 resulted in significantly lower stiffness than prone (*p* < 0.05).

Male stiffness was also strongly influenced by posture during relaxation (L3: F = 19.98, *p* < 0.001; L4: F = 16.57, *p* < 0.001; L5: F = 12.60, *p* < 0.001) and contraction (L3: F = 6.75, *p* = 0.003; L4: F = 4.00, *p* = 0.026; L5: F = 4.12, *p* = 0.024). During relaxation, both sitting and standing consistently produced significantly higher stiffness across L3–L5 compared to prone (*p* < 0.05). However, during contraction, standing at L3 was associated with significantly lower stiffness compared to prone (*p* < 0.05), mirroring the pattern observed in females.

Regarding tone, posture significantly affected female tone across all levels during both relaxation (L3: F = 8.64, *p* = 0.001; L4: F = 6.33, *p* = 0.004; L5: F = 5.06, *p* = 0.010) and contraction (L3: F = 13.35, *p* < 0.001; L4: F = 11.34, *p* < 0.001; L5: F = 6.74, *p* = 0.003). Post hoc analysis indicated that sitting consistently produced significantly higher tone across L3, L4, and L5 in both states compared to prone (*p* < 0.05). Conversely, standing during contraction resulted in significantly lower tone across all levels compared to prone (*p* < 0.05).

For males, posture significantly affected tone during relaxation across all levels (L3: F = 14.64, *p* < 0.001; L4: F = 11.59, *p* < 0.001; L5: F = 8.40, *p* = 0.001), with both sitting and standing resulting in significantly higher tone at L3–L5 than prone (*p* < 0.05). However, male tone during contraction was significant only at L3 (F = 3.50, *p* = 0.040), driven by the sitting posture producing significantly higher tone than prone (*p* < 0.05), whereas posture effects at L4 (F = 2.53, *p* = 0.09) and L5 (F = 2.57, *p* = 0.09) were not statistically significant.

### 3.4. Segmental Differences

Significant differences among spinal levels (L3–L5) were observed for female damping, stiffness, and tone in the prone posture (both relaxed and contracted states) and in standing during relaxation (*p* < 0.05, Table 3). In general, females exhibited higher values at the upper lumbar segment (L3) compared with L4 and L5. In contrast, male damping, stiffness, and tone showed no significant differences among levels across any posture or muscle state (*p* > 0.05), indicating a more uniform segmental profile in males.

## 4. Discussion

This study revealed that the biomechanical properties of the LES muscle, namely damping, stiffness and muscle tone, are shaped by posture, gender, spinal segment and muscle contraction state. Posture exerted the largest and most consistent effects, with clearly significant main effects on all three properties, particularly on stiffness and muscle tone. The spinal segment showed significant main effects on damping, stiffness, and muscle tone, whereas gender had marked main effects on stiffness and muscle tone. The main effect of muscle contraction state alone was not significant. Still, significant interaction effects were observed between posture and gender, between posture and muscle contraction state, and higher-order interactions that combined posture, gender, and muscle contraction state. These findings indicate that the biomechanical properties of the LES results from the combined influences of body position, regional spinal level, gender differences and the state of muscle activation. Collectively, these findings indicate that LES biomechanical behavior arises from the combined influences of body position, regional spinal level, gender-related morphological differences, and activation state, rather than from any single factor in isolation.

Transitioning from a sitting to a standing posture induces substantial changes in the spine’s anterior–posterior curvature. Compared with standing, sitting reduces lumbar lordosis and produces a posterior pelvic tilt, thereby altering the sagittal spinal alignment [22,23]. Prior research indicates that when the spine deviates from its neutral alignment, muscle tension increases almost immediately [24]. Moreover, different sitting postures modulate the positioning and load distribution of individual spinal segments in distinct ways, thereby modifying spinal loading and the mechanical properties of the LES [23].

In the present study, posture exerted significant effects on all three mechanical properties of the LES, particularly on stiffness and muscle tone. In both males and females, stiffness and tone were higher in sitting and standing than in the prone position, with sitting consistently showing the highest values. Previous myotonometry studies similarly demonstrated that sitting increases LES stiffness to a greater extent than standing in healthy adults [23]. This suggests that in a seated posture, the lumbar extensor muscles must sustain greater mechanical demand to maintain spinal stability [25]. These findings align with earlier reports showing elevated muscle activation and passive stiffness of the LES in sitting and standing relative to prone lying, with sitting producing the most pronounced effects [17,23,26,27]. Such posture-induced increases in stiffness and tone may reflect heightened passive and reflexive tension as the musculature compensates for gravitational loading [28].

Notably, these posture-related elevations in stiffness and muscle tone carry important clinical implications, as they may serve as biomechanical indicators of potential muscular overload and imbalance. Prolonged sitting or reduced physical activity may facilitate the accumulation of metabolic by-products and sustained low-level activation, further elevating stiffness and tone in the seated position and predisposing the musculature to fatigue and compromised spinal stability [29]. In individuals with low back pain, this increase in stiffness has been shown to be more pronounced on the painful side [5], suggesting that asymmetrical stiffness may be a relevant marker of dysfunction. From a clinical monitoring perspective, posture-sensitive biomechanical assessment may complement radiographic and clinical examinations by enabling longitudinal tracking of muscular adaptation in growing children undergoing brace treatment for scoliosis, particularly in the context of growth-related changes and prolonged postural constraints [30]. In individuals with chronic low back pain, integrating posture-dependent biomechanical measures into multimodal, guideline-based management frameworks may facilitate more individualized follow-up and help inform adjustments to conservative treatment strategies [31].

Damping, on the other hand, represents the muscle’s ability to absorb and dissipate energy [1]. The present results indicate that damping values were lower in the standing posture, suggesting reduced viscous behavior, greater energy transmission efficiency, and decreased tissue compliance—conditions that may facilitate rapid postural adjustments. However, if low damping persists for prolonged periods, the reduced energy-absorption capacity may contribute to localized muscle fatigue and increased risk of microtrauma [1]. Overall, muscle tone, stiffness, and damping collectively reflect the tension loading, mechanical responsiveness, and energy-exchange capacity of the LES under different postures and activity states, all of which influence spinal stability and fatigue risk. Understanding how these biomechanical properties adapt across postures may help in preventing the development of chronic low back pain.

This study further revealed a significant interaction between posture and muscle contraction state, suggesting that muscle activation level and spinal alignment jointly modulate the mechanical properties of the LES, particularly stiffness and tone. In the relaxed condition, stiffness and tone were most significant in the sitting position, followed by standing, and lowest in the prone position. During voluntary contraction in standing and sitting, both parameters tended to decrease relative to the relaxed state, although this pattern was not consistent across all levels and genders. Notably, in standing, LES tone decreased during contraction compared to relaxation at certain lumbar segments. One plausible explanation is that, in a fully extended upright posture, part of the load may be transferred to passive structures, such as ligaments and fascia, thereby reducing the mechanical demand on the LES [4].

A particularly noteworthy observation was that in the standing posture, LES tone decreased during contraction. This counterintuitive pattern may be related to changes in muscle length and load redistribution that occur during active lumbar extension [32]. As the LES contracts, slight spinal extension shortens the muscle fibers, reducing passive viscoelastic tension and thereby lowering the oscillation frequency detected by the myotonometer. At the same time, coordinated activation of the abdominal muscles and lower limb stabilizing muscles in standing may redistribute mechanical load, reducing the passive contribution required from the LES. This pattern of coordinated activation among stabilizing muscles, including the abdominals, gluteals, and hip extensors, can enhance trunk stability and spinal stiffness by sharing load across passive structures and synergistic muscles [33,34]. Under such conditions, myotonometric measures of tone, which primarily reflect passive mechanical stiffness rather than neural drive [35], may decrease even when overall muscular effort and postural control demands increase. This finding highlights the need to interpret myotonometric measurements within the broader context of neuromuscular control strategies rather than equating tone directly with electromyography (EMG) activity.

Gender differences constituted another notable component of the findings. Males demonstrated consistently higher stiffness and tone across lumbar segments, corroborating previous observations [10,18]. This pattern suggests an inherent sexual dimorphism in the mechanical properties of the LES, likely attributable to physiological and structural differences between genders. Men typically possess greater muscle density, larger cross-sectional area, and higher muscle mass, as well as differences in connective tissue composition, collagen density, and overall body mass distribution [6,7,8,36,37]. These morphological differences are likely to increase baseline stiffness and tone in males, potentially conferring greater load-bearing capacity but also influencing fatigue patterns and injury susceptibility under repetitive loading.

The influence of the lumbar segmental level further refined the pattern of LES mechanical behavior. Women exhibited higher damping, stiffness, and muscle tone at the upper lumbar segment (L3), suggesting that this region may be more sensitive to variations in local anatomical configuration and biomechanical loading. These segment-specific differences may arise from regional variations in muscle architecture, moment arm length, and spinal curvature, which together influence mechanical leverage and load distribution along the spine [2,3]. In contrast, males did not show similar intersegmental differences, which may be attributable to their greater overall muscle mass and the relatively uniform load distribution along the spinal column [38,39]. Moreover, the segmental differences in females were more evident across postures and contraction states, particularly for damping. Under more relaxed conditions, such as prone or standing, females showed apparent segmental variations in damping, stiffness, and tone from L3 to L5, whereas males showed minimal segmental differences. These findings indicate that females exhibit greater segmental sensitivity to postural changes, consistent with prior evidence of gender-related influences on lumbar extensor activation patterns and trunk kinematics [40]. Clinically, these results underscore the importance of considering gender and segment-specific characteristics when developing assessment protocols and designing targeted interventions, particularly for female populations with lumbar dysfunction.

Several limitations of this study should be acknowledged. The sample consisted solely of healthy young adults, which restricts generalizability to clinical populations or older adults. The cross-sectional design also limits causal interpretation. Additionally, the myotonometry device cannot differentiate between active and passive stiffness components, nor can it assess deeper musculature, such as the multifidus. Contraction intensity was based on subjective submaximal effort rather than objective quantification using EMG or dynamometry, potentially introducing variability. Future studies incorporating EMG, imaging, or controlled loading paradigms would help clarify mechanisms underlying posture-dependent changes in LES properties. Longitudinal research may further elucidate how training, fatigue, or rehabilitation interventions influence these mechanical characteristics and their relevance to lumbar health.

## 5. Conclusions

This study demonstrates that the biomechanical properties of the lumbar erector spinae are strongly influenced by posture, with stiffness, tone, and damping differing across prone, sitting, and standing positions. Posture exerted the greatest effect on LES mechanics, while gender and lumbar segment also showed significant main effects, indicating that these factors should be considered when establishing reference values or comparing groups. Overall, these findings support the development of posture- and gender-specific normative data for the assessment of lumbar muscle function.

## Figures and Tables

**Table 1:** Demography of participants.

Gender	Item	Mean ± SD
Female (n = 16)	Age (years)	21.06 ± 2.02
	Weight (kg)	51.88 ± 5.03
	Height (m)	1.59 ± 0.06
	BMI	20.64 ± 1.73
Male (n = 14)	Age (years)	21.21 ± 1.85
	Weight (kg)	66.93 ± 10.03
	Height (m)	1.74 ± 0.05
	BMI	22.01 ± 2.47

**Table 2:** Factors influencing the biomechanical properties of the lumbar erector spinae muscle.

	Damping					Stiffness					Tone				
	*df*	MS	F	*p* Value	η^2^	*df*	MS	F	*p* Value	η^2^	*df*	MS	F	*p* Value	η^2^
Between-subject effects															
Gender	1	0.07	0.32	0.58	0.01	1	2,677,701.51	14.27	<0.0167	0.34	1	951.43	9.95	<0.0167	0.26
Error	28	0.22				28	187,676.94				28	95.58			
Within-subject effects															
Posture	1	1.10	10.81	<0.0167	0.28	2	1,198,413.69	64.69	<0.0167	0.70	2	399.75	48.34	<0.0167	0.63
Posture × Gender	1	0.67	6.54	<0.0167	0.19	2	17,149.59	0.89	0.41	0.03	2	52.93	5.96	<0.0167	0.18
Level	1	0.37	15.98	<0.0167	0.36	1	233,339.73	20.72	<0.0167	0.43	1	137.39	43.80	<0.0167	0.61
Level × Gender	1	0.10	4.32	0.03	0.13	1	15,670.37	1.39	0.26	0.05	1	3.35	1.07	0.33	0.04
Action	1	0.02	0.62	0.44	0.02	1	2692.81	0.17	0.69	0.01	1	1.35	0.19	0.66	0.01
Action × Gender	1	0.02	0.71	0.41	0.03	1	39,431.96	2.42	0.13	0.08	1	2.04	0.29	0.59	0.01
Level × Posture	3	0.06	5.97	<0.0167	0.18	2	6269.46	1.78	0.17	0.06	2	1.00	1.07	0.35	0.04
Level × Posture × Gender	3	0.02	2.05	0.12	0.07	2	3618.84	1.03	0.38	0.04	2	0.25	0.26	0.78	0.01
Action × Posture	1	0.05	0.92	0.37	0.03	1	1,029,744.48	39.79	<0.0167	0.59	1	290.26	32.07	<0.0167	0.53
Action × Posture × Gender	1	0.01	0.14	0.78	0.01	1	211,291.49	8.17	<0.0167	0.23	1	19.78	2.18	0.14	0.07
Level × Action	2	0.00	0.56	0.58	0.02	2	1649.79	1.46	0.24	0.05	2	0.18	0.38	0.64	0.01
Level × Action × Gender	2	0.04	7.02	<0.0167	0.20	2	2166.57	1.77	0.18	0.06	2	0.13	0.28	0.71	0.01
Level × Action × Posture	3	0.05	6.52	<0.0167	0.19	3	17,180.95	8.62	<0.0167	0.24	2	8.22	13.65	<0.0167	0.33
Level × Action × Posture × Gender	3	0.02	2.18	0.10	0.07	3	1165.23	0.58	0.60	0.02	2	0.50	0.82	0.45	0.03
Error (Level × action × posture)	83	0.01				71	1992.68				60	0.60			

MS: mean square.

**Table 3:** Differences in damping, stiffness, and tone of erector spinae muscles (L3–L5) in different postures.

Myoton	Gender	Level	Prone		Sitting		Standing		^a^ Differences Among Postures			
			Relaxation	Contraction	Relaxation	Contraction	Relaxation	Contraction	Relaxation		Contraction	
									F	*p*	F	*p*
Damping (logarithmic decrement)	Female	L3	1.06 ± 0.13	1.08 ± 0.13	1.01 ± 0.08	1.01 ± 0.12	0.97 ± 0.08 *	1.00 ± 0.10	3.49	<0.05	2.13	0.13
		L4	0.98 ± 0.15	1.01 ± 0.19	1.01 ± 0.08	0.98 ± 0.09	0.92 ± 0.10	0.90 ± 0.10	2.62	0.08	2.96	0.06
		L5	0.90 ± 0.20	0.93 ± 0.16	0.99 ± 0.11	0.93 ± 0.11	0.85 ± 0.14	0.82 ± 0.20 ^#^	3.32	0.05	2.13	0.13
^a^ Difference among levels			<0.05	<0.05	>0.05	>0.05	<0.05	<0.05				
	Male	L3	1.19 ± 0.30	1.12 ± 0.27	0.88 ± 0.13 *	0.93 ± 0.09 *	0.97 ± 0.18 *	0.95 ± 0.14 *	7.35	<0.001	4.44	<0.05
		L4	1.07 ± 0.30	1.15 ± 0.32	0.91 ± 0.15	0.91 ± 0.13 *	0.95 ± 0.18	0.94 ± 0.18 *	1.98	0.15	4.77	<0.05
		L5	0.96 ± 0.26	1.13 ± 0.32	0.90 ± 0.18	0.89 ± 0.19 *	0.95 ± 0.18	0.98 ± 0.13	0.29	0.75	3.77	<0.05
^a^ Difference among levels			>0.05	>0.05	>0.05	>0.05	>0.05	>0.05				
Stiffness (N/m)	Female	L3	265.31 ± 38.77	370.45 ± 68.95	461.83 ± 105.81 *	415.62 ± 95.57	333.22 ± 124.8 ^#^	279.37 ± 68.72 *^#^	16.91	<0.001	7.58	<0.001
		L4	242.29 ± 47.06	304.88 ± 95.07	418.03 ± 110.61 *	385.55 ± 110.19 *	315.00 ± 154.28 ^#^	234.26 ± 50.76 ^#^	9.79	<0.001	11.58	<0.001
		L5	218.78 ± 36.54	241.88 ± 62.01	376.48 ± 130.31 *	344.49 ± 114.4 *	269.44 ± 147.98 ^#^	210.02 ± 47.18 ^#^	7.74	<0.001	12.37	<0.001
^a^ Difference among levels			<0.05	<0.05	>0.05	>0.05	<0.05	>0.05				
	Male	L3	304.73 ± 59.54	563.75 ± 113.73	598.53 ± 127.65 *	541.05 ± 133.49	503.87 ± 165.60 *	364.84 ± 101.46 *^#^	19.98	<0.001	6.75	<0.001
		L4	280.23 ± 66.33	496.99 ± 129.04	584.30 ± 152.70 *	547.53 ± 169.54	476.13 ± 180.24 *	362.30 ± 122.93 ^#^	16.57	<0.001	4.00	<0.05
		L5	269.64 ± 57.57	464.12 ± 131.55	560.59 ± 172.21 *	535.91 ± 93.02	438.57 ± 195.38 *	334.55 ± 123.78 ^#^	12.60	<0.001	4.12	<0.05
^a^ Difference among levels			>0.05	>0.05	>0.05	>0.05	>0.05	>0.05				
Tone (Hz)	Female	L3	15.13 ± 0.93	17.16 ± 1.59	18.36 ± 2.37 *	17.00 ± 1.57	16.50 ± 2.85	14.87 ± 0.93 *^#^	8.64	<0.001	13.35	<0.001
		L4	14.32 ± 0.99	15.98 ± 1.45	17.14 ± 2.36 *	16.25 ± 1.66	15.49 ± 2.93	14.11 ± 0.95 *^#^	6.33	<0.001	11.34	<0.001
		L5	13.81 ± 0.97	14.92 ± 1.39	16.29 ± 2.62 *	15.59 ± 1.91	14.55 ± 2.73	13.70 ± 0.99 *^#^	5.06	<0.05	6.74	<0.001
^a^ Difference among levels			<0.05	<0.05	>0.05	>0.05	<0.05	>0.05				
	Male	L3	15.81 ± 1.49	19.72 ± 5.24	22.03 ± 3.61 *	20.32 ± 3.61	18.83 ± 3.54 *^#^	16.74 ± 1.92	14.64	<0.001	3.50	<0.05
		L4	15.48 ± 1.53	18.40 ± 4.82	21.40 ± 4.18 *	19.80 ± 3.94	18.02 ± 3.50 ^#^	16.56 ± 2.25	11.59	<0.001	2.53	0.09
		L5	14.95 ± 1.65	17.50 ± 4.24	20.53 ± 4.80 *	19.40 ± 4.32	17.38 ± 3.65	16.19 ± 2.40	8.40	<0.001	2.57	0.09
^a^ Difference among levels			>0.05	>0.05	>0.05	>0.05	>0.05	>0.05				

^a^: Data were analyzed using one-way analysis of variance (ANOVA). * Significant difference compared with the prone posture within the same muscle action state, as determined by Dunnett’s post hoc test (*p* < 0.05). ^#^ Significant difference between relaxation and contraction within the same posture and spinal level, as determined by paired *t*-test comparisons (*p* < 0.05).

## Data Availability

The data that support the findings of this study are available from the corresponding author upon request.

## Abbreviations

The following abbreviations are used in this manuscript:

BMI	Body Mass Index
EMG	Electromyography
LES	Lumbar erector spinae muscle
